# IAPT and the internet: the current and future role of therapist-guided internet interventions within routine care settings

**DOI:** 10.1017/S1754470X20000033

**Published:** 2020-04-08

**Authors:** Graham R. Thew

**Affiliations:** ^1^Department of Experimental Psychology, University of Oxford, Oxford, UK; ^2^Oxford Health NHS Foundation Trust, Oxford, UK

**Keywords:** digital psychological therapy, dissemination, IAPT, implementation, internet interventions, therapist training

## Abstract

Compared with the traditional face-to-face format, therapist-guided internet interventions offer a different approach to supporting clients in learning skills to manage and overcome mental health difficulties. Such interventions are already in use within IAPT (Improving Access to Psychological Therapies) and other routine care settings, but given their potential to deliver treatment more efficiently and therefore increase availability and access to evidence-based interventions, their use is likely to increase significantly over the coming years. This article outlines what is meant by therapist-guided internet interventions and why an online format is thought to be advantageous for clients, therapists, services, and communities more broadly. It reviews the current evidence in the context of common therapist beliefs about internet-based treatment. It aims to identify gaps where further research is required, particularly in relation to the broader implementation of these treatments in IAPT and other routine clinical services. Specifically, it emphasises the importance of choosing the right programmes, providing adequate therapist training in their use, and considering practical and organisational issues, all of which are likely to determine the success of implementation efforts.

**Key learning aims:**

1. To understand what therapist-guided internet interventions are and their potential advantages.
2. To understand the current evidence base for these interventions.
3. To learn where further research is needed with regard to both the interventions themselves, and to their broader implementation in IAPT.

## Introduction

A principal aim of psychological therapies is to help people develop new skills for dealing with emotional problems, that can be applied not only to their current difficulties, but also to potential problems or setbacks in the future. The sustained benefits arising from this learning are one of the reasons why psychological therapies tend to have a slightly stronger recommendation compared with medication in current guidance from the National Institute for Health and Care Excellence (e.g. NICE, 2009, 2013). Traditionally the way these treatments have been delivered is through face-to-face or telephone sessions. However, there is no reason to assume these are the only way that new skills can be learned, and advances in technology mean that there are now a number of online alternatives. The delivery of psychological therapies online has been an active area of research for the last 20 years (Andersson *et al.*, 2019).

This article aims to review what is known and what is still to be investigated regarding internet-based psychological interventions and their implementation in routine clinical services. It will consider the literature relevant to the most common therapist beliefs about this treatment format, and suggest directions for further research on the implementation of such programmes in Improving Access to Psychological Therapies (IAPT) services and other routine practice settings.

### What are therapist-guided internet interventions?

In general, internet interventions consist of online programmes that present therapy content similar to that used in face-to-face interventions (Teachman, 2014). The methods used to present content vary between programmes, but can include text from self-help interventions, slides in the form of a short presentation, audio and video material, and exercises to complete. Many, although not all, programmes include guidance from a trained professional. The type and extent of guidance varies between different programmes but most commonly takes the form of written messages sent to the client on a regular basis to provide feedback on the work they have completed on the programme, offer support and encouragement, and advise on how to proceed.

### Why is this format of therapy useful?

Delivering psychological therapies online is thought to have a number of potential benefits that can ultimately improve the accessibility and availability of evidence-based treatment (e.g. Fairburn and Patel, 2017). For patients, these include the flexibility of being able to work on their treatment at a time and location convenient to them, such as evenings and weekends, which may not be as practical for face-to-face appointments. Patients may therefore be better able to fit treatment around work or childcare commitments, and may appreciate not having to disclose information about their treatment to employers. By not having to attend face-to-face appointments, patients avoid the time and costs of travel, and could therefore use this time to work on the treatment. Patients may appreciate the ability to work through treatment at their own pace, and take opportunities to go back and review earlier sections of therapy, which also may be less practical in face-to-face sessions. Lastly, some programmes allow users to retain access to the treatment content for a period after therapy ends, which may be beneficial in consolidating learning from therapy and preventing relapse.

For therapists, services, and communities more broadly, the main advantage is that less therapist time may be required to support a patient through a course of treatment. The potential is therefore for significant increases in treatment efficiency and cost-effectiveness, meaning that more people can be treated leading to further improvements in access to evidence-based therapies. Because much of the content of internet interventions is fixed within a programme and therefore delivered consistently, they are thought to be able to increase the ‘reach’ of psychological therapy: this would include more rural and remote areas, and following relevant translation and/or cultural adaptation, to people from countries and cultures from across the world (e.g. Fairburn and Patel, 2014). In its first ten years, the IAPT programme in England has made remarkable progress in making psychological therapies for common mental health problems more accessible to those who need them, but even now is reaching fewer than one in five of those in the community who are experiencing mental health difficulties (Clark, 2018). The global picture is similar, with less than one-third of those experiencing mental health problems receiving any form of treatment, and most of this treatment in the form of medication (Ormel *et al*., 2008). Training more therapists is essential for increasing access, but the impact of expanded training can potentially be magnified by the use of therapist-guided internet interventions.

## Internet interventions in IAPT

Internet interventions have been in use in IAPT since it started, predominantly offering CBT-based programmes as a Step 2 treatment. For therapist-guided interventions the guidance is often provided by psychological wellbeing practitioners who have received training in the specific programme being offered. A 2017 review of the use of mental health web and smartphone apps within the NHS highlighted that of the 191 IAPT services that provided information, 88.5% reported recommending or using online interventions (including apps) as part of their service provision, although of these, only 24.3% were using a NICE-recommended programme (Bennion *et al*., 2017). The results highlighted that the use of internet interventions was inconsistent across the country, and that many of the specific websites and apps being used and recommended have not undergone robust clinical evaluation. Data from the 2016–17 IAPT Annual Report showed that while guided self-help interventions were common, representing about 22% of all treatment, the vast majority of these were provided in book format, with only 12% delivered by computer (NHS Digital, 2017).

Looking ahead to the next 10 years of IAPT, it is likely that internet interventions will form a growing proportion of service provision, and that providing online psychological therapy will feature more commonly within IAPT professionals’ caseloads. Further research into the effectiveness of programmes used in IAPT is needed, and examples of this are already underway (Richards *et al*., 2018a). The use of therapist-guided internet interventions to deliver high-intensity therapy content is likely to expand. For example in social anxiety disorder (SAD) and post-traumatic stress disorder (PTSD), where the recommendation by NICE is for treatment at Step 3, online therapist-guided programmes to deliver the full content of recommended Step 3 cognitive therapy protocols now exist, have shown promising outcomes in initial studies (Stott *et al*., 2013; Thew *et al*., 2019; Wild *et al*., 2016), and are now being piloted in IAPT services.

Scaling up and providing broader implementation of these treatments requires careful consideration of the factors that may affect this process. This includes furthering our understanding of the most effective ways to deliver them, and how to integrate them within services. For therapist-guided internet interventions, just as in face-to-face therapy, it is important that therapists feel confident and motivated to provide treatment in this format. However, as this may be a new approach for many professionals, there are some common beliefs and concerns about this treatment format (Bengtsson *et al*., 2015; Donovan *et al*., 2015; Kivi *et al*., 2015; Meisel *et al*., 2018), for which it may be helpful to review the available literature.

## Commonly held beliefs

### ‘Internet-based therapies don’t have a strong evidence base’

Providing psychological therapy online is often perceived to be a fairly recent development that therefore may have limited empirical evidence of efficacy. However, this approach has now been in use for over 20 years, and there are around 300 controlled trials examining their efficacy (Andersson *et al*., 2019), mainly in the treatment of anxiety and depression. There are several things that can be concluded from the literature to date. First, it is clear from a number of systematic reviews, metaanalyses and a recent Cochrane review that online therapies (online CBT is the most frequently studied) show consistently superior outcomes compared with no treatment (Andrews *et al*., 2018; Kampmann *et al*., 2016; Olthuis *et al*., 2016). However, the quality of some of the individual studies within these reviews has been rated as low, meaning further high-quality studies would be beneficial. Second, there is good evidence to suggest that people achieve better outcomes from programmes with therapist support than those without it (Andersson and Cuijpers, 2009; Baumeister *et al*., 2014), which it has been suggested may result from better treatment adherence (Mohr *et al*., 2011). Third, a significant proportion of studies have included longer-term follow-up assessments up to 5 years post-treatment (e.g. Hedman *et al*., 2014; Johansson *et al*., 2017; Mörtberg *et al.*, 2011). A recent review of 14 such studies found there is good evidence that the clinical effects of treatment are sustained over this time (Andersson *et al*., 2018). Overall, it can therefore be concluded that there is now a strong evidence base for internet-based therapies, although given the range of interventions and delivery formats, further rigorous studies are necessary to support the use of specific online programmes. This field remains a highly active research area, meaning the number of empirical evaluations will continue to grow rapidly.

### ‘Internet-based therapies won’t be as effective as face-to-face therapy’

Another common viewpoint is that even if internet-based therapies may show some benefit, these are unlikely to be as effective as traditional face-to-face therapy. There are far fewer studies directly comparing guided online therapies with face-to-face interventions, compared with those investigating internet-based therapies in isolation. The results of these studies have generally found no substantial differences between these treatment formats, suggesting they may be equally effective, and this conclusion was supported by the results of a meta-analysis of 20 studies investigating treatments for a range of clinical problems (Carlbring *et al*., 2018). Similar conclusions have been drawn in other reviews (Andrews *et al*., 2018; Olthuis *et al.*, 2016). However, the quality of some of the individual studies has again been questioned, and it may be true that some of these comparison studies have not been sufficiently rigorous. In particular, it needs to be ensured that the face-to-face treatments being used as a comparison are being delivered to the highest possible standards, for example through the involvement of experts or the treatment developers as therapists or supervisors, and the measurement of therapist competence. It is also true that some internet interventions have high rates of drop-out, or low take-up, when contrasted with face-to-face therapies, and it would be helpful to see more detailed reporting of this, along with examinations of why this occurs and how to prevent it. Lastly it should be noted that like other therapies, online programmes may have potential negative effects, such as distress due to realizing the extent of a problem or being unable to complete a therapy task, or an overall worsening of symptoms that should be carefully monitored and reported (Rozental *et al*., 2014; Rozental *et al*., 2015). Overall the literature therefore suggests that internet interventions may achieve efficacy comparable to face-to-face therapy, although further robust studies are needed.

### ‘The therapeutic alliance won’t be as strong online’

One potential concern among professionals is that it may not be possible to develop a therapeutic alliance when working online, or that this would not be as strong compared with face-to-face, and that this may therefore limit engagement and outcomes of treatment. Studies have examined the question of online therapeutic alliances using standardized measures such as the Working Alliance Inventory (Horvath and Greenberg, 1989), along with qualitative studies of therapist emails, or interviews with clients. These studies have shown that clients can and do report strong working alliances with their therapist (Andersson *et al*., 2012b; Hadjistavropoulos *et al*., 2017; Kiluk *et al*., 2014; Knaevelsrud and Maercker, 2007; Perera-Delcourt and Sharkey, 2019; Schneider *et al*., 2016), and that these are in line with the strength of those reported for face-to-face interventions (Andersson *et al*., 2012b; Knaevelsrud and Maercker, 2007). Providing questionnaire feedback (i.e. observing changes and suggesting what these signify), praising and reinforcing activities completed by the client, and sending encouraging personalised messages have been associated with stronger perceptions of the therapeutic alliance (Perera-Delcourt and Sharkey, 2019; Schneider *et al*., 2016). However, it is possible that the nature of the working alliance may be different online; there is some evidence to suggest it is less strongly linked to clinical outcome compared with face-to-face therapy (Andersson *et al*., 2012b; Hadjistavropoulos *et al*., 2017). It is possible that the client’s ability to send a message to their therapist at any time may provide a perceived sense of continued support. It has also been suggested that clients may develop a working alliance not just with the therapist, but also with the programme itself (Kiluk *et al.*, 2014), which may warrant further investigation.

### ‘Internet-based therapies are only suitable for less severe problems’

Another common perception is that internet interventions would only be suitable for problems of mild to moderate severity. This may be understandable given that these programmes may most commonly be offered as a low-intensity intervention, and that some research studies target milder problems specifically. However, there is evidence from studies of depression to suggest that people with severe problems can still achieve good outcomes (Richards *et al*., 2018b), and that across a range of low-intensity interventions (including but not limited to online programmes), initial depression severity was not associated with outcome (Bower *et al*., 2013). Furthermore, an individual patient data meta-analysis found that clients with severe symptoms may benefit more (Karyotaki *et al*., 2018). It is also noted that clients do not appear to hold this view; people attending an Australian internet-based treatment clinic were found to be just as severe (regarding symptoms, distress, and functional impact) as those attending a face-to-face clinic (Titov *et al*., 2010a). The number of studies investigating severity is currently limited, and it should be noted that many studies exclude participants with severe symptoms if there is elevated risk of self-harm or suicide, given this can be more difficult to manage when working online. At present the literature indicates it may be possible to use internet interventions to treat severe problems successfully, although it would be helpful for this to be examined further in future studies. It may therefore be appropriate to focus more on other factors that may determine client suitability, for example level of risk, client availability, treatment preference, and therapy goals.

### ‘Internet-based therapies will fail to meet clients’ expectations of therapy’

One further belief held by some professionals is that an intervention in online format will not meet clients’ expectations of psychological therapy and may therefore not be perceived as acceptable. As outlined above, there is now extensive evidence of efficacy for internet-based therapies, which would not have been obtained without a reasonably high level of acceptability to clients. Many studies also report high levels of client satisfaction with treatment, although demand effects, and the influence of clinical outcome on satisfaction may limit these findings. It is also the case that many studies recruit participants from the community via advertising, which could result in samples who are generally more interested and positive about internet interventions. There are relatively few studies examining clients’ beliefs about and experiences of internet-based therapies. Participants of such studies generally describe their treatment experiences as positive, feeling it was beneficial overall and feeling supported by the therapist, but note the demanding nature of treatment, and difficulties finding the time and motivation to work on the programme (Halmetoja *et al*., 2014; Richards *et al*., 2016). One qualitative study highlighted that clients may have initial negative expectations and beliefs about online therapy, suggesting that clear information about the programme and what to expect needs to be provided (Perera-Delcourt and Sharkey, 2019). Several of the participants in this study found that treatment then exceeded their expectations. However, it is also true that common to all therapy, some people have negative experiences. One study found that for some participants whose initial expectations were high, the iCBT for depression programme examined was perceived as disappointing (Bendelin *et al*., 2011). Overall it is therefore difficult to draw conclusions given the variation between different programmes in terms of content, therapist guidance, and how they are introduced and used within services. There are examples where not meeting expectations has been the case, and further work to examine potential negative effects of this treatment format has been recommended (Rozental *et al*., 2014). However, there are also examples where online treatments exceed expectations and are perceived positively. Acceptability and satisfaction are generally reported to be high, but further work within routine practice settings would be beneficial.

## Directions for further research and evaluation

### What is the optimal type of therapist support?

Therapist-guided internet interventions can vary quite considerably with regard to the methods, frequency, and nature of the guidance provided. Currently in research trials and IAPT settings, therapist guidance is most often provided by a trained coach or psychological wellbeing practitioner, and commonly takes the form of written guidance sent on a once-weekly basis, that provides feedback on the work the client has completed, information on how to proceed (e.g. the next module), and encouragement to keep working through the programme. Some other programmes have been designed to incorporate more intensive therapist guidance, for example using more frequent therapist communication, or with brief telephone calls alongside regular messaging. Such programmes permit a greater level of tailored clinical input, for example helping the client to plan specific behavioural experiments to address their individual concerns, or supporting the client to address specific areas of avoidance within a written trauma narrative (e.g. the internet-based cognitive therapy programmes for SAD and PTSD described in Stott *et al*., 2013; Thew *et al*., 2019; Wild *et al*., 2016; and the Interapy programme for PTSD described in Knaevelsrud and Maercker, 2007). It is likely that for some clinical presentations this more extensive support may be particularly advantageous. However, it may not be necessary in all circumstances. Importantly, some studies show that good outcomes can still be obtained with little or no therapist guidance (e.g. Boettcher *et al*., 2012; Titov *et al*., 2010b) and that additional support may not always lead to better outcomes (Berger *et al*., 2011). Further research is required to examine the impact of different methods and frequencies of therapist communication, and to understand when more intensive support would be most useful.

There is also a question regarding who should provide the guidance. In the case of the internet cognitive therapy programmes for SAD and PTSD mentioned above, the content of these treatments are the same as high-intensity face-to-face interventions, in line with NICE guidance for these conditions. As the therapist’s role is therefore to support the effective delivery of high-intensity content, then it can be argued this should be done by someone trained as a high-intensity therapist. However, it may of course be possible for the guidance to be provided by a trained low-intensity therapist. While some studies have compared treatment outcomes when the guidance was provided by professionals of different levels of qualification, and found no significant differences (e.g. Andersson *et al*., 2012a), this has yet to be examined in relation to programmes that involve more extensive or clinically informed guidance.

### How can we successfully implement these therapies at scale in IAPT and other routine clinical services?

Implementing internet-based therapies more broadly has real potential to provide further improvements to access and allow more people to receive treatment. IAPT has an excellent track record in, and infrastructure for, the dissemination of evidence-based treatments. So given the potential benefits to this treatment format described earlier, research and evaluation of these programmes within IAPT will allow investigation of whether these potential advantages can be realised.

There is evidence to suggest that internet-based therapies can be successfully implemented within routine practice settings, and achieve outcomes similar to those from randomised controlled trials (Andersson and Hedman, 2013; Andrews *et al*., 2015; Nordgreen *et al*., 2018; Titov *et al*., 2018). However, studies of effectiveness in routine care are less common compared with those from research contexts, so more are needed to understand how they work in practice in greater depth. It is possible that evidence-based internet interventions developed in research contexts may not bridge the gap into routine practice, and a number of potential barriers to this have been identified, including lack of training opportunities, professionals’ reservations, and demonstrating cost-effectiveness (Gunter and Whittal, 2010; Weisz *et al*., 2014).

It is suggested that understanding and evaluating effective implementation requires the consideration of three key areas. Firstly we need to choose the right interventions. Given the amount and variety of online programmes and apps available, it is crucial to consider which will be appropriate for delivery in routine clinical settings. For IAPT settings, the key criteria to review are (1) whether the treatment content is NICE compliant; (2) whether the programme meets all NHS information governance and data security requirements; and (3) whether the programme is easy to use, for example an intuitive user interface for clients and therapists. Programmes must meet all three of these criteria to ensure they are evidence based, safe and secure, and practical, so that clients feel confident to use them, knowing how and where their data are used, and so that therapists can efficiently support, manage and communicate with those they are treating. NICE has been conducting preliminary independent evaluations along these lines for some programmes to date, but the same approach is recommended for other organisations and services when evaluating which programmes to use.

Second, we need to ensure adequate therapist training to ensure the programmes are implemented as intended so as to achieve optimal results. This means that the therapists learning to use the programme develop clear knowledge of the specific treatment protocol and structure, understand how to use the programme at a technical level and the methods used for communicating with clients, learn what the therapist’s role is and what the content of their communication should cover (both of which may be quite different compared with face-to-face therapy), and practise strategies to deal with common problems such as low client motivation or limited engagement. For example, one skill is being able to refer clients back to specific earlier sections of the programme if it might be useful to recap something, rather than delivering this content directly (as would be done in face-to-face work). Such training will require access to suitable training cases, and regular supervision, particularly in the early stages. In cases where an internet intervention is closely based on a specific face-to-face therapy protocol, it may be helpful for therapists to be provided with refresher training on the face-to-face treatment, in order to have a clear sense of what each online therapy component is trying to achieve. Further research evaluating therapist training for internet interventions is much needed, which should include examination of the best methods to provide training, the assessment of therapist competence in their delivery, and evaluation of how training can be scaled up within the NHS (for example through a ‘train-the-trainer’ model, or the use of online training programmes).

Thirdly, we need to think carefully about the practical and organisational factors relevant to the delivery of these interventions within services. Having good programmes and well-trained therapists is not sufficient to ensure successful implementation if thought is not given to how the treatments will be integrated within routine service provision. The choice of staff to provide the guidance should be considered with respect to level of qualification, working pattern, and clinical capacity. It may be appropriate for therapists to have regular time slots protected for their internet-based work. To date, most studies of internet interventions in routine practice have taken place in specialist clinics where most or all treatment is provided online. As a result, less is known about how best to effectively combine face-to-face and online service provision. Supervision arrangements for online work require additional consideration given that the clinical approach can differ from face-to-face work. In terms of the technical aspects, arrangements need to be made for how to enrol clients into a programme, how technical support is provided, and how therapists’ clinical activity should be recorded (for example, integration with existing electronic case management systems).

Lastly, one of the main considerations for implementation is how online service provision fits within existing referral pathways, and the stepped care model in IAPT. Internet interventions that deliver low-intensity content are commonly offered as an initial treatment option, with the ability to step to another treatment if this does not lead to improvement. For internet interventions delivering high-intensity content (for example, some programmes for SAD and PTSD) it is suggested that these should also be considered as a first treatment option, primarily given that NICE recommends high-intensity content as the first-line treatment, but also because clients may find it more acceptable to begin with an online treatment, before being ‘stepped up’ to face-to-face work if required, rather than the reverse.

## Conclusions

Treating psychological problems using therapist-guided internet interventions has significant potential to increase the availability and accessibility of evidence-based treatment. The empirical basis for this treatment format is substantial and appears to be more extensive than is commonly thought. However, there remain unanswered questions that require further research, in particular developing our understanding of how they perform in routine clinical practice. IAPT offers significant scope to examine this question, although implementation is unlikely to be straightforward and requires careful consideration of which interventions to use, who the therapists should be and how they should be trained, and how internet-based therapies will integrate into the service as a whole. However, the story of IAPT’s first ten years is that by continually considering such factors and putting appropriate procedures in place, recovery rates have improved from 40 to 52% (Clark, 2018). We may assume, then, that internet treatments will follow a similar path and that it may take time to embed these new programmes into services, with outcomes increasing as this process happens. The next ten years will bring exciting opportunities for clinicians, researchers and services to implement and evaluate a range of internet interventions, and it is hoped this will help us make progress towards achieving the significant potential that they offer.

## Key practice points

1. Therapist-guided internet interventions have a strong evidence base and are likely to become more widely used in IAPT and other routine clinical services over the coming years.
2. The therapist’s role in these interventions is different from face-to-face work and will therefore require some additional training specific to each programme.
3. Further research and evaluation of these treatments as part of routine service provision is required to understand their effectiveness in these settings, and how they should be best implemented.

